# Hepatic iron storage is related to body adiposity and hepatic inflammation

**DOI:** 10.1186/s12986-017-0169-3

**Published:** 2017-02-13

**Authors:** Chan Yoon Park, Jayong Chung, Kyung-Ok Koo, Min Soo Kim, Sung Nim Han

**Affiliations:** 1 Department of Food and Nutrition, College of Human Ecology, Seoul National University, 1 Gwanak-ro, Gwanak-gu, Seoul, South Korea; 2 Department of Food and Nutrition, College of Human Ecology, Kyung Hee University, Seoul, South Korea; 3 Research Institute of Human Ecology, Seoul National University, Seoul, South Korea

**Keywords:** Hepatic non-heme iron, Mild calorie restriction, Iron absorption, Duodenal iron transporter, Hepcidin, Body adiposity

## Abstract

**Background:**

Obesity has been reported to be associated with iron deficiency. However, few studies have investigated iron status in low adiposity. To investigate whether body adiposity was associated with altered hepatic iron status, we compared liver iron levels and markers involved in inflammation and iron absorption in obese, control, and mildly calorie restricted mice.

**Methods:**

Seven week old C57BL/6 mice were fed control (10% kcal fat, Control) or high fat (60% kcal fat, HFD) diets, or reduced amount of control diet to achieve 15% calorie restriction (CR) for 16 weeks. Hepatic non-heme iron content and ferritin protein level, and hematocrit and hemoglobin levels were determined to assess iron status. Hepatic expression of *Mcp-1* and *Tnf-α* were measured as hepatic inflammatory markers. Hepatic hepcidin (*Hamp*) and *Bmp6*, and duodenal *Dmt1*, *Dcyt1b*, hephaestin (*Heph*) and ferroportin mRNA levels were measured as factors involved in regulation of iron absorption.

**Results:**

Hepatic non-heme iron and ferritin protein levels were significantly higher in the CR group compared with the Control group, and significantly lower in the HFD group. These two iron status markers showed significantly negative correlations with the amount of white adipose tissue (*r* = -0.689 for hepatic non-heme iron and *r* = -0.740 for ferritin). Hepatic *Mcp-1* and *Tnf-α* mRNA levels were significantly lower in the CR compared with the HFD (74 and 47% lower) and showed significantly negative correlations with hepatic non-heme iron levels (*Mcp-1*: *r* = -0.557, *P* < 0.05; *Tnf-α*: *r* = -0.464, *P* < 0.05). Hepatic *Hamp* mRNA levels were lower in the HFD and higher in the CR groups compared with the Control group, which could be a response to maintain iron homeostasis. Duodenal *Dcyt1b* mRNA levels were higher in the CR group compared with the HFD group and duodenal *Heph* mRNA levels were higher in the CR group than the Control group.

**Conclusion:**

We showed that body adiposity was inversely correlated with liver iron status. Low inflammation levels in hepatic milieu and enhanced expression of duodenal oxidoreductases induced by calorie restriction could have contributed to higher iron status.

## Background

Iron deficiency is the most prevalent single nutrient problem and the most common cause of anemia. More than two billion people in both developing and industrialized countries are anemic due to iron deficiency [1]. Iron deficiency and obesity are often observed together and obesity has been reported as a possible factor leading to under-nutrition of iron in recent years [2–8]. Although the etiology of iron deficiency associated with obesity is uncertain, many animal and clinical studies have reported reduced iron storage with obesity. Systemic chronic inflammation induced by obesity and higher hepcidin levels have been suggested as a reason for impaired iron absorption due to inhibition of iron transport across the basolateral enterocyte membrane by the action of hepcidin [3, 4, 9].

Hepcidin (*Hamp*) is the central regulator of iron homeostasis and is mainly released from the liver. When the iron level is high, iron activates the bone morphogenetic protein (BMP) pathway and phosphorylates Smad to increase hepcidin expression. Hepcidin binds to ferroportin and induces its internalization, preventing efflux of iron from enterocyte to the circulation [10–12]. When the iron level is low, release of hepcidin from liver decreases [13]. Inflammatory states can also increase hepcidin expression. Lipopolysaccharide (LPS)-treatment increased *Hamp* mRNA levels in primary hepatocyte, Hep3B, and rat liver tissue [14, 15]. Obese patients with upregulated levels of serum C-reactive protein (CRP) and interleukin (IL)-6 had higher serum hepcidin levels [4, 5, 16]. Therefore, increased hepcidin expression mediated by inflammatory response has been suggested as one of the mechanisms for obesity related low iron level [11, 17, 18]. On the other hand, Sonnweber et al. [19] reported that iron deficiency observed with obesity was independent of hepcidin expression. Rather, it was suggested that decreased expression of iron absorption related duodenal enzymes, such as duodenal cytochrome B (Dcyt1b) and hephaestin, could affect iron deficiency in obesity. Other studies have reported that inflammatory markers, tumor necrosis factor alpha (TNF-α) and interferon gamma (IFN-γ), directly disturbed iron absorption into the enterocyte [4, 19–21].

Excess adiposity leads to chronic inflammatory responses by increasing macrophage infiltration into adipocytes. Growing evidence suggests that inflammation is one of the main contributors to iron deficiency [22, 23]. Calorie restriction has been reported to ameliorate inflammation through suppressing inflammatory cytokines. Monocyte chemoattractant protein 1 (*Mcp-1)*, *Il-6* and *Tnf-α* mRNA and CRP protein levels in adipose tissue were lowered in rodents with 30% or more calorie restriction compared with those fed ad libitum [24, 25]. However, iron status was not measured in these calorie restricted animals. If chronic inflammation caused by excess adiposity is linked to iron deficiency, calorie restriction may have the potential to change iron status. Therefore, studies are required to examine whether alleviation of inflammation and further reduction of body weight achieved by calorie restriction has an impact on iron status.

This study compared hepatic iron levels using mildly calorie restricted, obese, and control mice to clarify the relationship between adiposity and iron status. To understand the mechanism responsible for differences in iron storage associated with body adiposity, the levels of hepcidin, pro-inflammatory markers, and duodenal enzymes involved in iron transport were determined.

## Methods

### Animals and diets

Male C57BL/6 N mice (7 weeks old) were purchased from Central laboratory Inc. (Seoul, Korea) and individually housed in the specific pathogen free (SPF) animal facility at Seoul National University. After 1 week of acclimation with the control diet, mice were assigned to one of 3 dietary groups and fed experimental diets for 16 weeks: calorie restriction (CR, *n* = 16), control (Control, *n* = 24), and high fat (HFD, *n* = 25) diets. The Control group was fed the control diet (10% energy from fat, Research Diets, New Brunswick, NJ, USA, #D12450B) ad libitum and CR group was fed a reduced amount of control diet to achieve 15% calorie restriction. The HFD group was fed with a high fat diet (60% energy from fat, Research Diets, #D12492) ad libitum. After 16 weeks, mice were fasted for 12 h and euthanized by CO_2_ asphyxiation. Blood, liver, duodenum and white adipose tissue were collected and samples were stored at -80 °C.

### Determination of liver non-heme iron content

Hepatic non-heme iron levels were measured using the colorimetric method as previously described by Brain et al. [26]. Liver samples (100 mg) were homogenized and hydrolyzed in 2 ml of acidic solution (mixture of 3 mol/L HCl and 10% trichloroacetic acid). After 20 h incubation, supernatant was collected and mixed with chromogen reagent containing bathophenanthroline sulfonate. After 10 min incubation, absorbance was measured at 535 nm. Iron concentration was expressed as microgram of iron per gram of wet tissue.

### Determination of liver protein oxidation

Hepatic protein oxidation was determined using the Oxyblot™ Protein Oxidation Detection kit (S7150; Millipore, Billerica, MA, USA) based on Western blot analysis. Protein oxidation was detected by measuring carbonyl groups in the protein side chain derived from the oxygen free radicals. Briefly, liver protein was extracted and derivatized to 2, 4-dinitrophenylhydrazone (DNP-hydrazone), separated by SDS-PAGE, then transferred onto PVDF membrane. Membranes were incubated with rabbit anti-DNP (1:150) to detect DNPH-derivatized proteins, washed, then incubated with goat anti-rabbit IgG (1:300). After the reaction with chemiluminescent reagent, DNPH derivatized proteins were detected and analyzed using a mixture of standard proteins as the loading control.

### RNA preparation and real time PCR

RNA was extracted using TRIZOL reagent (Invitrogen, CA, USA) from liver and duodenum. Reverse transcription was performed using the PrimeScript™ 1st strand cDNA synthesis kit (Takara bio Inc., Japan). mRNA levels of *Hamp, Mcp-1, Tnf-α* in liver*, Fpn, Dmt1, Dcyt1b,* and *Heph* in duodenum were determined by real-time PCR with a SYBR Premix Ex Taq (Takara bio Inc.) and StepOne Real-time PCR System (Applied Biosystems, CA, USA). The relative expression of each gene was calculated from 2^-ΔΔCT^. All values were normalized to the levels of *Gapdh* and expressed as relative mRNA level compared to the average level of the Control group.

### Western blot analysis

Protein was isolated from liver tissue using RIPA lysis buffer and protein concentration was determined by Bradford assay (Bio-Rad Laboratories, CA, USA). Fourty μl of protein was separated by electrophoresis on a 10% SDS-polyacrylamide gel. Separated proteins were blotted onto a PVDF membrane and blocked in Tris buffered saline/0.1% Tween-20 (TBST, pH 7.6), containing 5% skim milk. Membrane was incubated with sheep anti-human ferritin (The Binding site Ltd., Birmingham, UK), washed, then incubated with HRP-conjugated donkey anti-sheep IgG (Santa Cruz, CA, USA). Membrane bound antibodies were visualized with enhanced chemiluminescence (ECL) solution (Santa Cruz). Band density was analyzed using QuantityOne software (Bio-Rad Laboratories) with β-actin as the loading control.

### Statistical analysis

One-way analysis of variance (ANOVA) was used to determine overall differences among groups, followed by Fisher’s least significant difference (LSD) test for individual group comparisons. Pearson’s correlation was used to determine the association between parameters. The results from all comparisons were considered significant at *P* < 0.05. Data were reported as mean ± SEM. All data were analyzed using the SPSS 21.0 program (SPSS Inc., IL, USA).

## Results

### Body weight and adipose tissue weight

After 16 weeks of feeding, body weights were significantly different among groups (*P* < 0.001). Body weight of the HFD group (48.80 ± 0.49 g) was higher than those of the Control (37.08 ± 0.56 g) and CR (27.53 ± 0.76 g) groups, and the CR group had lower body weight than the Control group. White adipose tissue weight was lower in the CR group than the Control (55% lower, *P* < 0.001) and HFD (74% lower, *P* < 0.001) groups, and it was lower in the Control group than the HFD group (43% lower, *P* < 0.001). Liver weight per body weight (g liver weight/100 g body weight) was also different among groups, and significantly lower in the CR group compared with the Control (*P* < 0.01) and HFD (*P* < 0.001) groups (Table 1).

**Table 1:** Body weight and weight gain of mice in the CR, Control, and HFD groups^1^

	CR (*n* =16)	Control (*n* = 24)	HFD (*n* = 25)	*P* value
Body weight at 0 week (g)	22.68 ± 0.20	22.40 ± 0.22	22.26 ± 0.21	0.44
Body weight at 16 week (g)	27.53 ± 0.76^a^	37.08 ± 0.56^b^	48.80 ± 0.49^c^	<0.001
Body weight gain (g)	4.8 ± 0.8^a^	14.7 ± 0.5^b^	26.5 ± 0.5^c^	<0.001
Daily calorie intake (kcal/day)	11.43 ± 0.16^a^	13.49 ± 0.14^b^	15.59 ± 0.15^c^	<0.001
White adipose tissue (g) ^2^	1.76 ± 0.14^a^	3.94 ± 0.15^b^	6.87 ± 0.11^c^	<0.001
Liver weight per body weight (g/100 g body weight)	4.18 ± 0.15^a^	5.11 ± 0.11^b^	5.58 ± 0.23^b^	<0.001

^1^One-way ANOVA followed by Fisher’s LSD multiple comparison was used to determine significant differences

^2^White adipose tissue weight included epididymal, subcutaneous, retroperitoneum, and perinephric fat

^abc^Different superscript letters indicate significant differences at *P* < 0.05

### Serum hemoglobin and hematocrit levels

Hemoglobin and hematocrit levels were not different among groups (Table 2).

**Table 2:** Levels of hemoglobin and hematocrit of mice in the CR, Control, and HFD groups^1^

	CR (*n* =16)	Control (*n* = 24)	HFD (*n* = 25)	*P* value
Hemoglobin (g/dl)	14.66 ± 0.21	14.42 ± 0.13	14.28 ± 0.21	0.409
Hematocrit (%)	49.31 ± 1.12^a^	47.01 ± 0.92^b^	46.92 ± 0.56^c^	0.124

^1^One-way ANOVA followed by Fisher’s LSD multiple comparison was used to determine significant differences

^abc^Different superscript letters indicate significant differences at *P* < 0.05

### Liver non-heme iron concentrations and ferritin protein levels

**Fig. 1:**
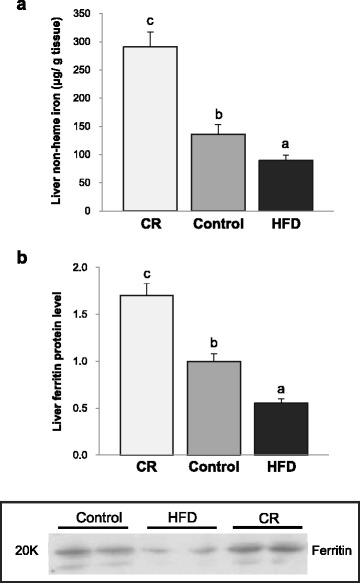
Liver non-heme iron concentration and ferritin protein level: **a** Liver non-heme iron level per gram of tissue (μg/g tissue); **b** Densitometric analysis of liver ferritin protein expression and representative ferritin Western blots. The intensity of ferritin was densitometrically measured and normalized to the protein expression level of β-actin. ^abc^Different superscript letters indicate *P* < 0.05. One-way ANOVA followed by Fisher’s LSD multiple comparison was used to determine significant difference. *n* = 16–25 per group

**Fig. 2:**
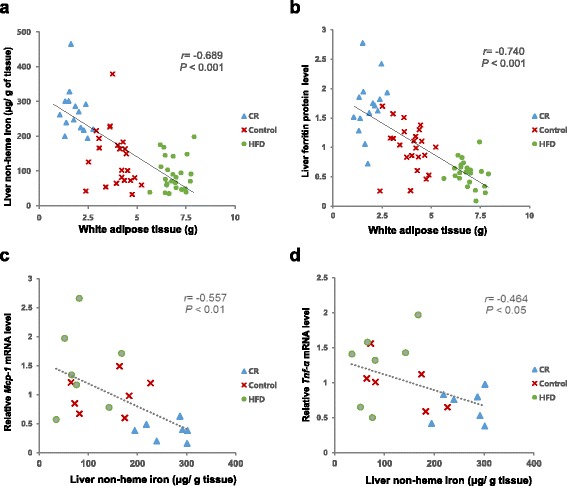
Correlations between liver iron levels and white adipose tissue or hepatic inflammatory markers: **a** Liver non-heme iron level (μg/g tissue) and white adipose tissue weight (g), *n* = 63; **b** Liver ferritin protein level and white adipose tissue weight (g), *n* = 63; **c** Hepatic *Mcp-1* mRNA level and non-heme iron level (μg/g tissue), *n* = 21; **d** Hepatic *Tnf-α* mRNA level and non-heme iron level (μg/g tissue), *n* = 21; Pearson correlation coefficient, r, and *P* value are indicated for each graph

Hepatic non-heme iron concentrations were significantly different among groups (*P* < 0.001). The CR group had significantly higher non-heme iron level (291. 20 ± 26.48 μg/g tissue) compared with the Control (136.21 ± 16.99 μg/g tissue) and HFD (89. 92 ± 9.52 μg/g tissue) groups (Fig. 1a). Hepatic ferritin protein levels were higher in the CR group than the Control (70% higher, *P* < 0.001) and HFD (207% higher, *P* < 0.001) groups. The HFD group’s ferritin level was significantly lower than that of the Control group (45% lower, *P* < 0.001) (Fig. 1b). Hepatic non-heme iron levels correlated negatively with white adipose tissue weights (*r* = -0.689, *P* < 0.001), and body weights *(r* = -0.711, *P* < 0.001) (Fig. 2a). Hepatic ferritin protein levels also showed negative correlations with white adipose tissue amount (*r* = -0.740, *P* < 0.001), and body weights (*r* = -0.708, *P* < 0.001) (Fig. 2b).

### Hepcidin (*Hamp*) and bone morphogenic protein *(Bmp6)* mRNA levels in liver

**Fig. 3:**
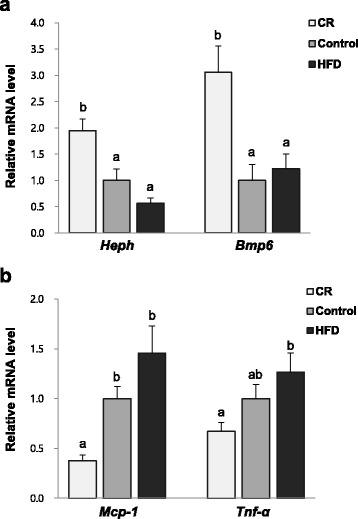
Relative hepatic mRNA levels of genes involved in iron homeostasis and inflammation: **a** *Heph* (*n* = 16–23 per group) and *Bmp6* (*n* = 7 per group) mRNA levels; **b** *Mcp-1* and *Tnf-a.* mRNA levels (*n* = 7 per group). Data are presented as mean ± SEM. ^ab^Different letters indicate *P* < 0.05. One-way ANOVA followed by Fisher’s LSD multiple comparison was used to determine significant difference. All values were normalized to the levels of housekeeping gene *Gapdh* and expressed as relative mRNA level compared to the average level of the Control group

Hepatic *Hamp* mRNA levels were significantly different among groups. The CR group had higher *Hamp* mRNA levels than the Control (95% higher) and HFD (242% higher) groups and the Control group had higher mRNA levels than the HFD group (76% higher, *P* = 0.073) (Fig. 3a). The mRNA levels of *Bmp6* which regulates hepcidin expression were higher in the CR group compared with the Control (206% higher) and HFD (151% higher) groups. There was no significant difference in *Bmp6* mRNA levels between Control and HFD groups (Fig. 3a). Hepatic *Hamp* and *Bmp6* mRNA levels showed positive correlations with non-heme iron levels (*Hamp: r* = 0.600, *P* < 0.001; *Bmp6: r* = 0.707, *P* < 0.001, data not shown). On the other hand, negative correlations were observed between white adipose tissue weights and hepatic *Hamp* and *Bmp6* mRNA levels (*Hamp: r* = -0.567, *P* < 0.001; *Bmp6: r* = -0.559, *P* < 0.01, data not shown).

### Pro-inflammatory cytokines mRNA levels in liver

Figure 3b shows hepatic *Mcp-1* and *Tnf-a* mRNA levels. *Mcp-1* mRNA levels were lower in the CR group compared with other two groups (63% lower than Control, *P* < 0.05 and 74% lower than HFD, *P* < 0.001). *Tnf-a* mRNA levels were lower in the CR group than the HFD group (47% lower, *P* < 0.05). The mRNA levels of *Mcp-1* and *Tnf-a* negatively correlated with liver iron concentration (*r* = -0.557, *P* < 0.01; *r* = -0.464, *P* < 0.05, respectively) (Fig. 2c and d).

### Oxidation status of proteins in liver

**Fig. 4:**
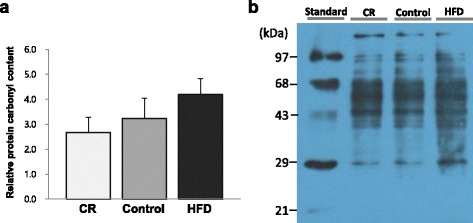
The oxidation status of liver proteins: **a** Densitometric analysis of protein carbonyls; **b** Representative Western blots for protein carbonyls in the four lines of standard protein and liver protein in CR, Control, and HFD groups. The intensity of protein carbonyls was densitometrically measured and normalized by the level of standard protein. Data are presented as mean ± SEM, *n* = 7 for each group. One-way ANOVA followed by Fisher’s LSD multiple comparison was used to determine significant difference

Hepatic protein oxidation levels were not significantly different among groups (Fig. 4).

### Iron transporters, oxidoreductases and ferritin mRNA levels in duodenum

**Fig. 5:**
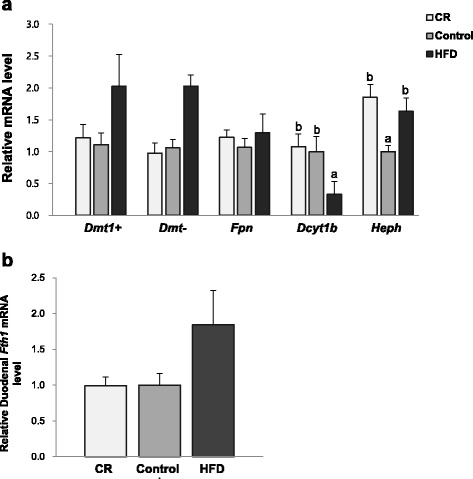
Relative duodenal mRNA levels of ferritin and enzymes involved in iron transport: **a** *Dmt1*, *Fpt(ferroportin)*, *Heph*(*hephaestin)*, and *Dcyt1b* mRNA levels; **b** *Fth1(ferritin)* mRNA levels. Data are presented as mean ± SEM, *n* = 7–12 for each group. ^ab^Different letters indicate *P* < 0.05. One-way ANOVA followed by Fisher’s LSD multiple comparison was used to determine significant difference. All values were normalized to the levels of housekeeping gene *Gapdh* and expressed as relative mRNA level compared to the average level of the Control group

To investigate whether HFD induced obesity and calorie restriction affected iron absorption, mRNA levels of duodenal enzymes involved in iron transport, divalent metal transporter1 (*Dmt1*), ferroportin (*Fpn*), duodenum cytochrome 1b (*Dcyt1b*), and hephaestin (*Heph*) were measured (Fig. 5). mRNA levels of *Dmt1* and *Fpn* were not significantly different among groups. However, oxidoreductase *Dcyt1b* expression was significantly higher in the CR (229% higher) and Control (204% higher) groups compared with the HFD group. *Heph* expression was significantly higher in the CR (85% higher) and HFD (63% higher) groups compared with the Control group. There was no significant difference in duodenal ferritin *(Fth1*) mRNA levels among groups.

## Discussion

The present showed that liver iron levels were inversely associated with body weights and white adipose tissue amount. The study showed not only that hepatic iron storage was lower in the HFD group, which is consistent with previous findings [3], but also that lower body weight achieved by mild calorie restriction resulted in higher non-heme iron concentration and hepatic ferritin protein level in comparison with Control animals. Inflammation in hepatic milieu and levels of duodenal oxidoreductases appear to be involved in the adiposity related differences in liver iron status.

To achieve mild calorie restriction, the CR group were fed 15% reduced amount of control diet without additional supplements. Iron levels of the experimental diets were 35 mg Fe/kg in the control diet, the NRC suggested requirement for iron [27], and 48 mg Fe/kg in the high fat diet. Considering daily diet intake of each group, actual average iron intake was 104 μg Fe/day in the CR group, 15% lower than that in the Control group (123 μg Fe/day). Sorbie and Valberg [28] reported that diets containing 25–100 mg Fe/kg diet could support normal growth and hematopoiesis of C57BL/6 J mice. Therefore, a 15% decrease in dietary iron intake is unlikely to affect normal growth and metabolism.

Despite a lower dietary iron intake, hepatic non-heme iron levels were 114% higher and ferritin protein levels were 60% higher in the CR group compared with the Control group. On the other hand, the HFD group showed 34% lower non-heme iron levels and 45% lower ferritin protein levels compared with the Control group, although their iron intake was 16% higher than the Control group. Human and animal studies have also reported reduced iron status with obesity [3, 4, 29]. Restoration of serum iron and hemoglobin levels after weight loss in obese people has been observed in several studies, which suggest an association between adiposity and iron status [30, 31]. Higher iron status in very lean mice from the CR group compared with the control animals further supports the close association between adiposity and iron levels.

Obesity could lead to chronic inflammation and oxidative stress; due to macrophage recruitment into adipose tissue and release of pro-inflammatory cytokines through various signals. Reactive oxygen species (ROS) production is also increased by these cytokines and overconsumption of oxygen during obesity [32–34]. On the other hand, calorie restriction has been reported to alleviate inflammation and ROS production [24, 35, 36]. In this study, mRNA levels of *Tnf-α* and *Mcp-1* showed positive correlations with white adipose tissue amount, and expression of these cytokines were significantly higher in the HFD group than the CR group. However, degrees of oxidative stress, examined by determination of protein oxidative carbonyl content, were not significantly different among the groups. According to the review by Dixon et al. [37], iron and iron derivatives were essential for ROS producing enzyme function and they contributed to formation of ROS. Higher non-heme iron and ferritin levels in the CR group could have led to higher redox-active iron pools which would contribute to the ROS formation. Therefore, lower inflammation in the CR group did not result in significant reduction of oxidative stress.

Not only hepatic iron concentration but also hepatic ferritin protein levels, which are both involved in iron storage, showed negative correlations with hepatic *Mcp-1* and *Tnf-α* levels. TNF-α has been reported to induce hypoferremia by inhibiting iron absorption through the small intestine in mice [20, 38]. *Mcp-1* has been reported as a TNF-α induced gene [39]. Although the mechanism has not been explicitly elucidated, Laftah et al. [38] reported that mucosal transfer of iron was reduced in TNF-α treated mice and was associated with increased level of duodenal ferritin that could trap ferric iron within the enterocyte. In this study, although not statistically significant (*P* = 0.102), mean duodenal *Fth1* mRNA level was higher in the HFD group compared with the Control and CR groups, which was opposite to the case of ferritin protein levels in liver. In this regard, increased *Tnf-α* and *Mcp-1* expression could be responsible for decreased iron absorption throughout the enterocyte.

Many previous studies that reported iron deficiency with obesity suggested that hepcidin was responsible for obesity driven iron shortage and hepcidin was upregulated by inflammatory signals [9]. However, in the present study, the mRNA levels of *Hamp* and *Bmp6* were lowest in the HFD group and highest in the CR group. Hepcidin is the main regulator of systemic iron homeostasis by reducing total iron content and its expression is regulated by Bmp6 [12, 17]. Hepcidin synthesis is increased by excess iron levels or inflammatory signals and decreased by iron deficiency, hypoxia, or erythroid [40]. Darshan et al. [14] showed that inflammation induced by LPS increased *Hamp* expression in normal mice, while this mechanism was blunted in LPS stimulated mice with iron deficiency, as their *Hamp* levels were decreased. They concluded that severe iron deficiency could ameliorate hepcidin expression in response to LPS. In this study, although *Mcp-1* and *Tnf-α* mRNA levels were higher in the HFD group, indicative of increased inflammatory response, hepcidin levels were lower than the other groups. Lower iron levels in the HFD group could have overridden the effect of these inflammatory mediators on hepcidin expression. Thus, hepcidin levels seemed to be mainly regulated by bodily requirements for iron in this study.

Expression of iron absorption related enzymes in duodenum tissue was determined to investigate the association between adiposity and iron status. mRNA levels of *Dcyt1b* and *Heph*, which is located at both sides of enterocyte and facilitate iron transport, were highest in the CR group. Dcyt1b is the ferric reductase that converts ferric iron to the ferrous state that enables iron to enter the apical side of enterocyte through the Dmt1, and hephaestin is the ferroxidase at the basolateral side of the enterocyte [41, 42]. Calorie restriction could have promoted iron absorption by upregulating those duodenal enzymes that enable the physical state of iron to enter the enterocyte. Sonnweber et al. [19] reported lowered *Dcyt1b* and *Heph* mRNA levels in obese mice compared with control mice. They suggested that decreased levels of ferroxidases were associated with iron deficiency in obesity. In this study, *Dcyt1b* expression was lowest in the HFD group and it showed significant negative correlation with white adipose tissue amount (*r* = -0.509), however, hephaestin level was upregulated in the HFD group compared with the Control group. Considering that hephaestin is affected by stored iron amount rather than by instant dietary iron intake [41], low iron status in the HFD group could have influenced *Heph* expression and led to higher expression in the HFD group. However, further research is needed to identify the relationship between adiposity and duodenal iron absorption and to find obesity related factors that regulate expression of iron transport enzymes.

## Conclusions

This study confirmed that liver iron status has an inverse relationship with body adiposity. More importantly, we showed that liver iron levels could be upregulated in low adiposity through mild calorie restriction. Low inflammatory state induced by calorie restriction could be responsible for higher hepatic iron storage. Upregulated duodenal iron transport enzymes appeared to contribute to higher hepatic iron levels in the CR group.

## Abbreviations

ANOVA: One-way analysis of variance
BMP: Bone morphogenetic protein
CR: Calorie restriction
CRP: C-reactive protein
DCYT1B: Duodenum cytochrome 1b
DMT1: Divalent metal transporter1
ECL: Enhanced chemiluminescence
HFD: High fat diet
IFN-γ: Interferon gamma
IL-6: Interleukin-6
LPS: Lipopolysaccharide
LSD: Least significant difference
MCP-1: Monocyte chemoattractant protein 1
ROS: Reactive oxygen species
SPF: Specific pathogen free
TNF-α: Tumor necrosis factor alpha
